# Supplementary material for: Anti-Methanogenic Effect of Phytochemicals on Methyl-Coenzyme M Reductase—Potential: In Silico and Molecular Docking Studies for Environmental Protection
Source: Micromachines (Basel). 2021 Nov 19;12(11):1425. doi: 10.3390/mi12111425 (PMC8617884; doi:10.3390/mi12111425)
Supplement: Supplementary file 1 [file micromachines-12-01425-s001.zip › micromachines-1387081-supplementary.pdf]

micromachines

MDPI

Supplementary Material

Anti-methanogenic Effect of Phytochemicals on Methyl-Coenzyme M Reductase—Potential: In Silico and Molecular Docking Studies for Environmental Protection

Yuvaraj Dinakarkumar [1,*,†], Jothi Ramalingam Rajabathar [2,*,†], Selvaraj Arokiyaraj [3,†], Iyyappan Jeyaraj [1], Sai Ramesh Anjaneyulu [1], Shadakshari Sandeep [4], Chimatahalli Shanthakumar Karthik [4], Jimmy Nelson Appaturi [5] and Lee D. Wilson [6,*]

1 Vel Tech High Tech Dr. Rangarajan Dr. Sakunthala Engineering College, Anna University, Chennai-600062, Tamil Nadu, India; jiyyappan.biotek@gmail.com (I.J.); drsairamesh@gmail.com (S.R.A.)
2 Department of Chemistry, College of Sciences, P.O. Box 2455, King Saud University, Riyadh 11451, Saudi Arabia
3 Department of Food Science and Biotechnology, Sejong University, Seoul-05006, Korea
4 Department of Chemistry, S J College of Engineering, JSS Science and Technology University, Mysuru 570006, India; sandeep12chem@gmail.com (S.S.); csk@jssstuniv.in (C.S.K)
5 School of Chemical Sciences, Universiti Sains Malaysia, 11800 Penang, Malaysia; jimmynelson@usm.my
6 Department of Chemistry, University of Saskatchewan, Saskatoon, SK S7N 5C5, Canada
* Correspondence: yuvarajdinakarkumar@gmail.com (Y.D.); jrajabathar@ksu.edu.sa (J.R.R.); lee.wilson@usask.ca (L.D.W.), Tel.: +1-306-966-2961 (L.D.W.)
† Equally contributing authors

Citation: Dinakarkumar, Y.; Rajabathar, J.R.; Arokiyaraj, S.; Jeyaraj, I.; Anjaneyulu, S.R.; Sandeep, S.; Karthik, C.S.; Appaturi, J.N.; Wilson, L.D. Anti-Methanogenic Effect of Phytochemicals on Methyl-Coenzyme M Reductase–Potential: In Silico and Molecular Docking Studies For Environmental Protection. *Micromachines* **2021**, *12*, 1425. https://doi.org/10.3390/mi12111425

Academic Editors: Anna Vikulina

Received: 3 September 2021
Accepted: 10 November 2021
Published: 19 November 2021

Publisher's Note: MDPI stays neutral with regard to jurisdictional claims in published maps and institutional affiliations.

Copyright: © 2021 by the authors. Licensee MDPI, Basel, Switzerland. This article is an open access article distributed under the terms and conditions of the Creative Commons Attribution (CC BY) license (http://creativecommons.org/licenses/by/4.0/).

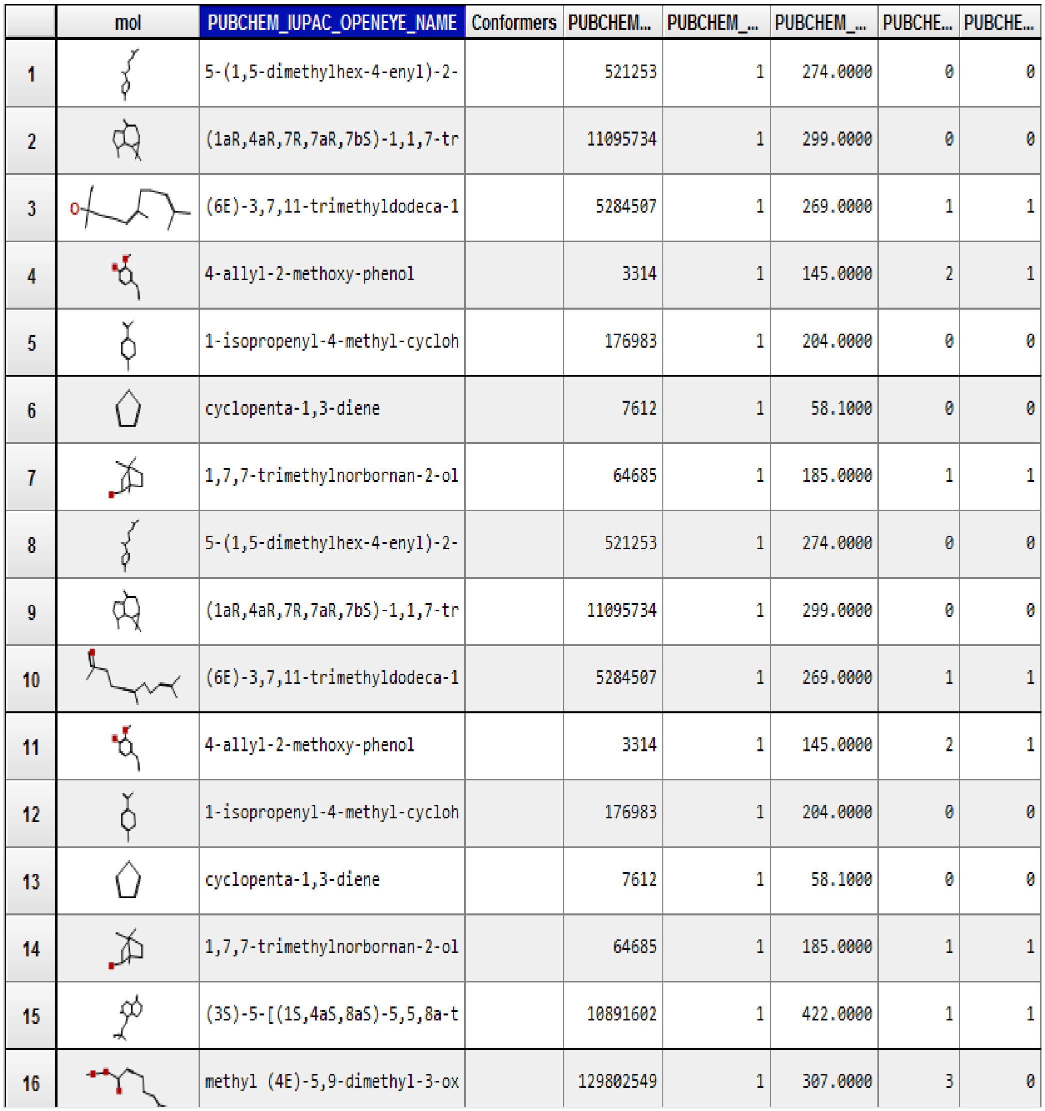

	mol	PUBCHEM_IUPAC_OPENEYE_NAME	Conformers	PUBCHEM...	PUBCHEM_...	PUBCHEM_...	PUBCHE...	PUBCHE...
1		5-(1,5-dimethylhex-4-enyl)-2-		521253	1	274.0000	0	0
2		(1aR,4aR,7R,7aR,7bS)-1,1,7-tr		11095734	1	299.0000	0	0
3		(6E)-3,7,11-trimethyldodeca-1		5284507	1	269.0000	1	1
4		4-allyl-2-methoxy-phenol		3314	1	145.0000	2	1
5		1-isopropenyl-4-methyl-cycloh		176983	1	204.0000	0	0
6		cyclopenta-1,3-diene		7612	1	58.1000	0	0
7		1,7,7-trimethylnorbornan-2-ol		64685	1	185.0000	1	1
8		5-(1,5-dimethylhex-4-enyl)-2-		521253	1	274.0000	0	0
9		(1aR,4aR,7R,7aR,7bS)-1,1,7-tr		11095734	1	299.0000	0	0
10		(6E)-3,7,11-trimethyldodeca-1		5284507	1	269.0000	1	1
11		4-allyl-2-methoxy-phenol		3314	1	145.0000	2	1
12		1-isopropenyl-4-methyl-cycloh		176983	1	204.0000	0	0
13		cyclopenta-1,3-diene		7612	1	58.1000	0	0
14		1,7,7-trimethylnorbornan-2-ol		64685	1	185.0000	1	1
15		(3S)-5-[(1S,4aS,8aS)-5,5,8a-t		10891602	1	422.0000	1	1
16		methyl (4E)-5,9-dimethyl-3-ox		129802549	1	307.0000	3	0

Figure S1. An illustrative example of the developed database for various ligand systems examined in this study.

Table S1. List of ligands (n = 166) selected for the study.

Compound Name	SMILE Format
(−) Zingiberene	CC1=CCC(C=C1)C(C)CCC=C(C)C
(−)-Spathulenol	CC1(C2C1C3C(CCC3(C)O)C(=C)CC2)C
(+)-Aromadendrene	CC1CCC2C1C3C(C3(C)C)CCC2=C
(E)-Nerolidol	CC(=CCCC(=CCCC(C)(C=C)O)C)C
(E,E)-*α*-Farnesene	CC(=CCCC(=CCC=C(C)C=C)C)C
(Z)-*β*-Farnesene	CC(=CCCC=CCC=C(C)C=C)C
1,3,4-Eugenol	COC1=C(C=CC(=C1)CC=C)O
1,3,8-p-Menthatriene	CC1=CC=C(CC1)C(=C)C
1,3-Cyclopentadiene	C1C=CC=C1
3,7-dimethyl-Endo-Borneol	CC1(C2CCC1(C(C2)O)C)C
13-epi-Manool	CC1(CCCC2(C1CCC(=C)C2CCC(C)(C=C)O)C)C
Methyl trans-geranylacetate	CC(=CCCC(=CC(=O)CC(=O)OC)C)C
2,4,7,9-Tetramethyl-5decyn4,7diol	CC(C)CC(C)(C#CC(C)(CC(C)C)O)O
2,6-Bis(3,4methylenedioxyphenyl) - 3,7-dioxabicyclo (3.3.0)octane	C1C2C(C(O1)C3=CC4=C(C=C3)OCO4)C(=O)OC2C5=C6C(=CC=C5)OCO6
2-Methyl-5-(1-propenyl)pyrazine	CC=CC1=NC=C(N=C1)C
4-Methyl-1-(1-methylethnyl) cyclohexene	CC1CCC(=CC1)C(=C)C
allo-Aromadendrene	CC1CCC2C1C3C(C3(C)C)CCC2=C
Citronellol	CC(CCC=C(C)C)CCO
β-Sitosterol	CCC(CCC(C)C1CCC2C1(CCC3C2CC=C4C3(CCC(C4)O)C)C)C(C)C
Diallyl tetrasulfide	C=CCSSSSCC=C
epi-Cubebol	CC1C=CC(C2C13C2C(CC3)(C)O)C(C)C
Eugenol	COC1=C(C=CC(=C1)CC=C)O
Farnesol	CC(=CCCC(=CCCC(=CCO)C)C)C
Geraniol	CC(=CCCC(=CCO)C)C
Geranylacetate	CC(=CCCC(=CCOC(=O)C)C)C
Limonene	CC1=CCC(CC1)C(=C)C
Linalool	CC(=CCCC(C)(C=C)O)C
Linalyl acetate	CC(=CCCC(C)(C=C)OC(=O)C)C
Nerol	CC(=CCCC(=CCO)C)C
p-Cymene	CC1=CC=C(C=C1)C(C)C
Phytol	CC(C)CCCC(C)CCCC(C)CCCC(=CCO)C
Pinacol	CC(C)(C(C)(C)O)O
Pulegone	CC1CCC(=C(C)C)C(=O)C1
Rosifoliol	CC1CCCC2(C1=CC(CC2)C(C)(C)O)C
Viridiflorol	CC1CCC2C1C3C(C3(C)C)CCC2(C)O
α-Santalene	CC(=CCCC1(C2CC3C1(C3C2)C)C)C
β-Pinene	CC1(C2CCC(=C)C1C2)C
τ-Cadinol	CC1=CC2C(CCC(C2CC1)(C)O)C(C)C
α-cadinol	CC1=CC2C(CCC(C2CC1)(C)O)C(C)C
α-Terpinyl acetate	CC1=CCC(CC1)C(C)(C)OC(=O)C
**TABLE S1:** continued	
α-Thujene	CC1=CCC2(C1C2)C(C)C
β-Caryophyllene	CC1=CCCC(=C)C2CC(C2CC1)(C)C
β-Cubebene	CC1CCC(C2C13C2C(=C)CC3)C(C)C
(E,E)- Farnesol	CC(=CCCC(=CCCC(=CCO)C)C)C

1,8-CINEOLE	CC1(C2CCC(O1)(CC2)C)C
1-epi-Cubenol	CC1CCC(C2C1(CCC(=C2)C)O)C(C)C
2-Methyl-benzoxazole	CC1=NC2=CC=CC=C2O1
2-Propenoic acid, 3-phenyl, 2-phenylethyl ester	C1=CC=C(C=C1)CCOC(=O)C=CC2=CC=CC=C2
2-Thujene	CC1C=CC2(C1C2)C(C)C
3-O-Acetyloleanolic Acid	CC(=O)OC1CCC2(C(C1(C)C)CCC3(C2CC=C4C3(CCC5(C4CC(CC5)(C)C)C(=O)O)C)C)C
4'5-DIHYDROXY-3',6,7- TRI-METHOXYFLAVONE	COC1=CC2=C(C=C1)C(=O)C(=C(O2)C3=CC(=C(C(=C3)OC)O)O)OC
5,4'-DIHYDROXY-6,7,8,3'- TETRA-METHOXYFLAVONE	COC1=C(C=CC(=C1)C2=CC(=O)C3=C(C(=C(C(=C3O2)OC)OC)OC)O)OC4C(C(C(C(O4)O)O)O)O)O
6-HYDROXY-LUTEOLIN	C1=CC(=C(C=C1C2=CC(=O)C3=C(O2)C=C(C(=C3O)O)O)O)O)O
6-Oxa-bicyclo[3.1.0] hexan-3-one	C1C2C(O2)CC1=O
8-METHOXY-CIRSILINEOL	COC1=C(C=CC(=C1)C2=CC(=O)C3=C(C(=C(C(=C3O2)OC)OC)OC)O)O
ACETYL-CHOLINE	CC(=O)OCC[N+](C)(C)C
Allicin	C=CCSS(=O)CC=C
Allyl-Mercaptan	C=CCS
Alpha-Amyrin	CC1CCC2(CCC3(C(=CCC4C3(CCC5C4(CCC(C5(C)C)O)C)C)C2C1C)C)C
Alpha-Linolenic-Acid	CCC=CCC=CCC=CCCCCCCCC(=O)O
Alpha-Pinene	CC1=CCC2CC1C2(C)C
Alpha-Terpinene	CC1=CC=C(CC1)C(C)C
Alpha-Tocopherol	CC1=C(C2=C(CCC(O2)(C)CCCC(C)CCCC(C)CCCC(C)C)C(=C1O)C)C
Apigenin	C1=CC(=CC=C1C2=CC(=O)C3=C(C=C(C=C3O2)O)O)O
Ar-Curcumene	CC1=CC=C(C=C1)C(C)CCC=C(C)C
Benzyl-Cinnamate	C1=CC=C(C=C1)COC(=O)C=CC2=CC=CC=C2
Beta-Carotene	CC1=C(C(CCC1)(C)C)C=CC(=CC=CC(=CC=CC=C(C)C=CC=C(C)C=CC2=C(CCCC2(C)C)C)C)C
Beta-Phellandrene	CC(C)C1CCC(=C)C=C1
Beta-Pinene	CC1(C2CCC(=C)C1C2)C
Beta-Sitosterol	CCC(CCC(C)C1CCC2C1(CCC3C2CC=C4C3(CCC(C4)O)C)C)C(C)C
Beta-Terpineol	CC(=C)C1CCC(CC1)(C)O
Bicyclo[3,1,1] heptanes, 6,6,-dime-thyl-2methylene	CC1(C2CCC(=C)C1C2)C
Biotin	C1C2C(C(S1)CCCCC(=O)O)NC(=O)N2
Cadalene	CC1=CC2=C(C=CC(=C2C=C1)C)C(C)C
Caffeic-Acid	C1=CC(=C(C=C1C=CC(=O)O)O)O
Campesterol	CC(C)C(C)CCC(C)C1CCC2C1(CCC3C2CC=C4C3(CCC(C4)O)C)C
Carnosol	CC(C)C1=C(C(=C2C(=C1)C3CC4C2(CCCC4(C)C)C(=O)O3)O)O
Carvacrol	CC1=C(C=C(C=C1)C(C)C)O
Carvenone	CC1CCC(=CC1=O)C(C)C
Carveol	CC1=CCC(CC1O)C(=C)C
Caryophyllene	CC1=CCCC(=C)C2CC(C2CC1)(C)C
Chlorogenic-Acid	C1C(C(C(CC1(C(=O)O)O)OC(=O)C=CC2=CC(=C(C=C2)O)O)O)O
Chrysanthenone	CC1=CCC2C(=O)C1C2(C)C
Chrysoeriol	COC1=C(C=CC(=C1)C2=CC(=O)C3=C(C=C(C=C3O2)O)O)O
Cinnamaldehyde-[E]	C1=CC=C(C=C1)C=CC=O
Cinnamic-Acid	C1=CC=C(C=C1)C=CC(=O)O
Cirsimaritin	COC1=C(C(=C2C(=C1)OC(=CC2=O)C3=CC=C(C=C3)O)O)OC

Citral	CC(=CCCC(=CC=O)C)C
Citronellol	CC(CCC=C(C)C)CCO
citronellyl formate	CC(CCC=C(C)C)CCOC=O
cubenene	C12C3C4C1=C5C2C3=C45
Cumene	CC(C)C1=CC=CC=C1
Cuminaldehyde	CC(C)C1=CC=C(C=C1)C=O
Delta-3-Carene	CC1=CCC2C(C1)C2(C)C
Delta-Cadinene	CC1=CC2C(CCC(=C2CC1)C)C(C)C
D-Limonene	CC1=CCC(CC1)C(=C)C
D-Pinene	CC1=CCC2CC1C2(C)C
Endolysin	C(CCN)CC(C(=O)O)N
Eruboside-B	CC1CCC2(C(C3C(O2)CC4C3(CCC5C4CC(C6C5(CCC(C6)OC7C(C(C(C(O7)CO)OC8C(C(C(C(O8)CO)O)OC9C(C(C(C(O9)CO)O)O)O)OC2C(C(C(C(O2)CO)O)O)O)O)O)C)O)C)C)OC1
Eucalyptol	CC1(C2CCC(O1)(CC2)C)C
Eugenol	COC1=C(C=CC(=C1)CC=C)O
E-β-Farnesene	CC(=CCCC(=CCCC(=C)C=C)C)C
Farnesene	CC(=CCCC(=CCC=C(C)C=C)C)C
Farnesol	CC(=CCCC(=CCCC(=CCO)C)C)C
Ferulic-Acid	COC1=C(C=CC(=C1)C=CC(=O)O)O
Flavone	CC1=C(OC2=C(C1=O)C=C(C=C2)C[NH3+])C3=CC=CC=C3.[Cl-]
Furan, tetra hydro-3-methyl	CC1CCOC1
Gallic-Acid	C1=C(C=C(C(=C1O)O)O)C(=O)O
Germacrone	CC1=CCC(=C(C)C)C(=O)CC(=CCC1)C
Herniarin	COC1=CC2=C(C=C1)C=CC(=O)O2
Hesperidin	CC1C(C(C(C(O1)OCC2C(C(C(C(O2)OC3=CC(=C4C(=O)CC(OC4=C3)C5=CC(=C(C=C5)OC)O)O)O)O)O)O)O)O
Isopulegol	CC1CCC(C(C1)O)C(=C)C
Isorosmanol	CC(C)C1=C(C(=C2C(=C1)C3C(C4C2(CCCC4(C)C)C(=O)O3)O)O)O
Isovaleraldehyde	CC(C)C1=C(C(=C2C(=C1)C3C(C4C2(CCCC4(C)C)C(=O)O3)O)O)O
Kaempferol	C1=CC(=CC=C1C2=C(C(=O)C3=C(C=C(C=C3O2)O)O)O)O
Lignin	CC(C(C1=CC2=C(C(=C1)OC)OC(C2CO)C3=CC(=C(C=C3)OC(CO)C(C4=CC(=C(C=C4)O)OC)OC5=C(C=C(C=C5OC)C(C(CO)OC6=C(C=C(C=C6)C7C8COCC8C(O7)C9=CC(=C(C(=C9)OC)O)OC)OC)OC1=C(C=C(C=C1)C(C(CO)OC1=C(C=C(C=C1)C=CCO)OC)O)OC)O)OC)O)O
Limonene	CC1=CCC(CC1)C(=C)C
Lupeol	CC(=C)C1CCC2(C1C3CCC4C5(CCC(C(C5CCC4(C3(CC2)C)C)(C)C)O)C)C
Luteolin	C1=CC(=C(C=C1C2=CC(=O)C3=C(C=C(C=C3O2)O)O)O)O
Myrcene	CC(=CCCC(=C)C=C)C
Myricetin	C1=C(C=C(C(=C1O)O)O)C2=C(C(=O)C3=C(C=C(C=C3O2)O)O)O
Naringenin	C1C(OC2=CC(=CC(=C2C1=O)O)O)C3=CC=C(C=C3)O
Niacin	C1=CC(=CN=C1)C(=O)O
Ocimene	CC(=CCC=C(C)C=C)C
o-eugenol	COC1=CC=CC(=C1O)CC=C
Oleic-Acid	CCCCCCCCC=CCCCCCCCC(=O)O
p-Cymene	CC1=CC=C(C=C1)C(C)C
p-Hydroxy-Benzoic- Acid	C1=CC(=CC=C1C(=O)O)O
Pinene	CC1=C2CC(C2(C)C)CC1

Quercetin	C1=CC(=C(C=C1C2=C(C(=O)C3=C(C=C(C=C3O2)O)O)O)O)O
Quercetin-3-O-beta-D-Glucoside	C1=CC(=C(C=C1C2=C(C(=O)C3=C(C=C(C=C3O2)O)O)OC4C(C(C(C(O4)CO)O)O)O)O)[O-]
Rosmadial	CC(C)C1=C(C2=C(C(=C1)C=O)C3(CCCC(C3C=O)(C)C)C(=O)O2)O
Rosmanol	CC(C)C1=C(C(=C2C(=C1)C(C3C4C2(CCCC4(C)C)C(=O)O3)O)O)O
Rosmaridiphenol	CC(C)C1=C(C(=C2C(=C1)CCC3C(C2=O)CCCC3(C)C)O)O
Rosmarinic-Acid	C1=CC(=C(C=C1CC(C(=O)O)OC(=O)C=CC2=CC(=C(C=C2)O)O)O)O
Rosmariquinone	CC(C)C1=CC2=C(C3=C(C=C2)C(CCC3)(C)C)C(=O)C1=O
Rutin	CC1C(C(C(C(O1)OCC2C(C(C(C(O2)OC3=C(OC4=CC(=CC(=C4C3=O)O)O)C5=C C(=C(C=C5)O)O)O)O)O)O)O)O
S- Allylmercaptocystein E	C=CCSSCC(C(=O)O)N
Sabinene	CC(C)C12CCC(=C)C1C2
Sabinol	CC(C)C12CC1C(=C)C(C2)O
Safrole	C=CCC1=CC2=C(C=C1)OCO2
Squalene	CC(=CCCC(=CCCC(=CCCC=C(C)CCC=C(C)CCC=C(C)C)C)C)C
Stearic-Acid	CCCCCCCCCCCCCCCCCC(=O)O
Stigmasterol	CCC(C=CC(C)C1CCC2C1(CCC3C2CC=C4C3(CCC(C4)O)C)C)C(C)C
Terpinolene	CC1=CCC(=C(C)C)CC1
Trans-Carveol	CC1=CCC(CC1O)C(=C)C
Trans-Pinocarveol	CC1(C2CC1C(=C)C(C2)O)C
Umbelliferone	C1=CC(=CC2=C1C=CC(=O)O2)O
Vanillic-Acid	COC1=C(C=CC(=C1)C(=O)O)O
Xanthophyll	CC1=C(C(CC(C1)O)(C)C)C=CC(=CC=CC(=CC=CC=C(C)C=CC=C(C)C=CC2C(=C C(CC2(C)C)O)C)C)C
Zingiberene	CC1=CCC(C=C1)C(C)CCC=C(C)C
α-Bulnesene	CC1CCC2=C(CCC(CC12)C(=C)C)C
α-Copaene	CC1=CCC2C3C1C2(CCC3C(C)C)C
α-Humulene	CC1=CCC(C=CCC(=CCC1)C)(C)C
α-Selinene	CC1=CCCC2(C1CC(CC2)C(=C)C)C
α-Terpineol	CC1=CCC(CC1)C(C)(C)O
β-Bourbonene	CC(C)C1CCC2(C1C3C2CCC3=C)C
β-carene	CC1(C2C1CC(=C)CC2)C
β-Chamigrene	CC1=CCC2(CC1)C(=C)CCCC2(C)C
β-Elemene	CC(=C)C1CCC(C(C1)C(=C)C)(C)C=C
β-Eudesmol	CC12CCCC(=C)C1CC(CC2)C(C)(C)O
β-Gurjunene	CC1CCC2C(C2(C)C)C3C1CCC3=C
β-Selinene	CC(=C)C1CCC2(CCCC(=C)C2C1)C
γ-Eudesmol	CC1=C2CC(CCC2(CCC1)C)C(C)(C)O
δ-Cadinene	CC1=CC2C(CCC(=C2CC1)C)C(C)C
δ-cadinol	CC1=CC2C(CCC(C2CC1)(C)O)C(C)C
